# Molecular Profile of Advanced Endometrial Cancer (FIGO III–IV) in a Polish Multicentre Cohort: Clinicopathological Characterisation and Treatment Implications

**DOI:** 10.3390/jcm15187051

**Published:** 2026-09-11

**Authors:** Wiktor Szatkowski, Aleksandra Dudek, Katarzyna Franczyk, Małgorzata Nowak-Jastrząb, Tomasz Kluz, Małgorzata Cieślak-Steć, Magdalena Śliwińska, Paweł Blecharz

**Affiliations:** 1 Maria Skłodowska-Curie National Research Institute of Oncology, Kraków Branch, 31-115 Kraków, Poland; wiktor.szatkowski@krakow.nio.gov.pl (W.S.); malgorzata.nowak@krakow.nio.gov.pl (M.N.-J.); 2 Institute of Medical Sciences, University of Rzeszów, 35-310 Rzeszów, Poland; aleksandra.kmiec@vp.pl (A.D.); katarzyna.aniela.franczak@gmail.com (K.F.); jtkluz@interia.pl (T.K.); 3 Maria Skłodowska-Curie National Research Institute of Oncology, Gliwice Branch, 44-102 Gliwice, Poland; malgorzata.cieslak-stec@gliwice.nio.gov.pl (M.C.-S.); magdalena.sliwinska@gliwice.nio.gov.pl (M.Ś.)

**Keywords:** endometrial cancer, FIGO III–IV, molecular profile, p53abn, dMMR, POLEmut, immunotherapy, POLE mutation

## Abstract

**Background/Objectives:** Advanced endometrial cancer (FIGO III–IV) is characterised by poor prognosis and a heterogeneous biological profile, and molecular classification enables treatment personalisation by identifying subtypes with distinct therapeutic targets. We aimed to characterise the molecular and histopathological features of FIGO III–IV cases in a Polish multicentre cohort and to discuss the resulting treatment implications. **Methods:** This retrospective multicentre study included 915 consecutive patients with endometrial cancer operated on between April 2022 and May 2025 at three oncology centres in south-eastern Poland. Molecular subtyping (POLEmut, p53abn, dMMR/MSI-H, NSMP) was performed using immunohistochemistry (IHC) and next-generation sequencing (NGS). FIGO stage was assigned according to the FIGO 2009 classification. **Results:** Among 888 patients with a known molecular subtype, FIGO III–IV cases accounted for 15.9% (*n* = 141). The p53abn subtype predominated (35.5%), followed by dMMR/MSI-H (26.2%), NSMP (24.1%), and POLEmut (5.7%). The proportion of p53abn increased with stage (I–II vs. III–IV, *p* < 0.001), whereas dMMR/MSI-H remained stable regardless of stage (*p* = 0.83). POLEmut was absent in FIGO IV (0/16; 95% CI 0.0–19.4%), which should be regarded as an exploratory observation requiring prospective validation. **Conclusions:** The molecular profile of advanced endometrial cancer may inform treatment strategy; the therapeutic implications presented here are descriptive and hypothesis-generating, as the study did not include survival data. The dMMR/MSI-H subtype identifies patients who may benefit from immunotherapy in accordance with current clinical indications, supporting routine MMR testing regardless of disease stage. Conversely, p53abn tumours point to the need for a more intensive treatment strategy, in line with current guidelines. The absence of POLEmut in FIGO IV is an exploratory observation requiring prospective validation. Treatment de-escalation in POLEmut FIGO IIIC remains subject to further clinical validation and requires individualised assessment after complete staging.

## 1. Introduction

Endometrial cancer (EC) is the most common gynaecological malignancy in Poland and Europe [1]. Most cases are diagnosed at an early stage; however, advanced disease (FIGO III–IV) represents a significant therapeutic challenge owing to poor prognosis and the need for multimodal treatment. In the cohort described here, FIGO III–IV cases accounted for 15.9% of patients with a known molecular subtype.

A breakthrough in understanding the biology of endometrial cancer came with the results of The Cancer Genome Atlas (TCGA) project, which identified four distinct molecular subtypes of endometrial cancer [2]. The subsequently developed ProMisE system enabled practical implementation of this classification into routine pathological diagnostics [3]. The classification comprises four subtypes: POLE mutation (POLEmut), mismatch repair deficiency (dMMR/MSI-H), abnormal p53 expression (p53abn), and no specific molecular profile (NSMP). The four ProMisE groups serve as practical surrogates for the TCGA categories: POLE-ultramutated, MSI-hypermutated, copy-number-high/p53-abnormal, and copy-number-low/NSMP tumours, respectively. Each subtype differs in prognosis, recurrence pattern, and treatment response [4].

Current ESGO/ESTRO/ESP guidelines incorporate molecular classification into risk stratification and adjuvant treatment planning [1]. However, data on the distribution of molecular subtypes in advanced endometrial cancer (FIGO III–IV), particularly in Central and Eastern European populations, remain limited [4,5]. Moreover, the role of the molecular profile in individualising therapeutic management in this patient group continues to evolve as new clinical data emerge.

The aim of this study was to characterise the molecular subtypes and their clinicopathological features in patients with FIGO III–IV disease identified from a Polish multicentre cohort comprising 915 consecutive cases of endometrial cancer. In addition, we assessed the potential treatment implications arising from the observed distribution of molecular subtypes in advanced-stage disease. The added value of this work lies in providing data on the distribution of molecular subtypes in advanced endometrial cancer in a Central and Eastern European population, together with quantitative findings in line with current recommendations for universal MMR testing. We also separately analysed an exploratory observation regarding the distribution of the POLEmut subtype between FIGO stage III and IV.

## 2. Materials and Methods

### 2.1. Study Design and Population

This retrospective multicentre study included 915 consecutive patients with endometrial cancer treated surgically between April 2022 and May 2025 at three oncology centres in south-eastern Poland: the Maria Skłodowska-Curie National Research Institute of Oncology, Kraków Branch; the Institute of Medical Sciences, University of Rzeszów; and the Maria Skłodowska-Curie National Research Institute of Oncology, Gliwice Branch. The analysis focused on 141 patients with FIGO III–IV disease and a known molecular subtype. FIGO stage was assigned according to the FIGO 2009 classification in effect throughout the recruitment period. Retrospective restaging according to the FIGO 2023 classification was not feasible owing to incomplete availability of the required pathological parameters in historical records. The analytical populations comprised: the full surgical cohort (*n* = 915), patients with a conclusive molecular subtype (*n* = 888), the advanced-stage subgroup with a known subtype (FIGO III–IV; *n* = 141), and the non-MC subset used for clinicopathological comparisons (*n* = 129).

### 2.2. Immunohistochemistry and Molecular Analysis

Immunohistochemistry (IHC) included assessment of MMR proteins (MLH1, MSH2, MSH6, PMS2) and p53 expression. Staining was performed on the BenchMark Ultra platform (Ventana, Roche Diagnostics, Tucson, AZ, USA) using OptiView and UltraView detection kits with anti-p53 antibodies (clone BP53-11, Ventana, Tucson, AZ, USA; or clone DO-7, Dako/Agilent, Glostrup, Denmark), applied according to centre-specific validated protocols. dMMR/MSI-H status was defined by loss of expression of at least one MMR protein on IHC; equivocal cases were reviewed by a pathologist with dedicated expertise in gynaecological pathology. p53abn was defined according to ProMisE and WHO 2020 criteria as: overexpression (>80% of tumour cells with strong nuclear staining), null phenotype (complete absence of nuclear staining with preserved internal control), or other non-wild-type patterns (cytoplasmic or weak diffuse/blush staining). In the context of ICI eligibility, the term dMMR/MSI-H is used in accordance with the RUBY and DUO-E trial registrations; the primary qualifying test in this cohort was MMR IHC. p53 IHC is a recognised, reliable surrogate for TP53 mutational status in endometrial cancer [6]. Genomic DNA was extracted from FFPE material after pathologist-guided selection of blocks with adequate tumour cell content, using the Maxwell^®^ RSC DNA FFPE Kit (Promega, Madison, WI, USA). POLE mutations were assessed by Sanger sequencing (exons 9, 11, 13, and 14) according to the pathogenicity criteria of León-Castillo et al. [7]. In selected cases with discordant or equivocal IHC findings, additional NGS was performed on the IonTorrent platform (Thermo Fisher Scientific, Waltham, MA, USA) using a targeted amplicon panel covering POLE, TP53, MLH1, MSH2, MSH6, and PMS2. Detected variants were classified according to ACMG/AMP guidelines using publicly available databases (ClinVar, VarSome); only pathogenic and likely pathogenic variants were included in molecular classification. Variants of uncertain significance (VUS) were not used for subtype assignment.

### 2.3. Molecular Classification

Tumours were classified hierarchically according to the ProMisE system: (1) POLEmut > (2) dMMR > (3) p53abn > (4) NSMP. Twelve tumours (8.5%) in the FIGO III–IV subgroup met the criteria for multiple-classifier (MC) status and were reported descriptively, excluded from clinicopathological comparisons between the four canonical subtypes. In the overall cohort (FIGO I–IV), tumours with overlapping molecular features were classified according to the ProMisE hierarchy (POLEmut > dMMR > p53abn > NSMP) and assigned to a single dominant subtype; they were not identified or reported separately as MC outside the advanced-stage subgroup. This approach reflects the descriptive focus of the present analysis on FIGO III–IV disease and is consistent with our previous MC analysis [8] and the literature [9,10,11]. The hierarchical assignment was used for analytical classification only and did not determine individual treatment decisions in this retrospective dataset.

### 2.4. Assessment of ER/PR Receptors

Assessment of oestrogen and progesterone receptors (ER/PR) was not performed routinely during the study period (2022–2025), which preceded the ESGO 2025 recommendations that, for the first time, incorporated ER/PR into endometrial cancer risk stratification [1]. This represents a limitation of the present study.

### 2.5. Statistical Analysis

Statistical analyses were performed using IBM SPSS Statistics v29.0 (IBM Corp., Armonk, NY, USA). Categorical variables were compared using Fisher’s exact test in 2 × 2 form for comparisons between stages and the Fisher–Freeman–Halton extension for 2 × 4 comparisons between the four subtypes. Fisher-type tests were used instead of the χ^2^ test because expected cell counts < 5 occurred in every comparison (FIGO IV subgroup, *n* = 16, and POLEmut subgroup, *n* = 8). Proportions are presented with 95% Wilson (score) confidence intervals, appropriate for small samples and cells with zero counts. For the molecular subtypes, the odds ratio (OR) for advanced stages (FIGO III–IV vs. I–II) was additionally calculated for each subtype versus all others combined, with a 95% confidence interval using the Woolf (logit) method. A two-sided *p* < 0.05 was considered statistically significant. Given the exploratory nature of the analyses, no correction for multiple comparisons was applied.

### 2.6. Ethical Considerations

The study was conducted in accordance with the Declaration of Helsinki and received a positive opinion from the Bioethics Committee of the Maria Skłodowska-Curie National Research Institute of Oncology in Warsaw, Poland (Opinion No. 6/2025, dated 9 January 2025). The analysis was based on anonymised clinicopathological data collected during routine clinical care and processed in accordance with the General Data Protection Regulation (GDPR).

## 3. Results

### 3.1. Cohort Characteristics

The cohort comprised 915 patients undergoing molecular profiling; a conclusive molecular subtype was obtained in 888 patients. Twenty-seven cases were excluded because molecular subtyping could not be completed owing to insufficient tumour tissue and/or inadequate DNA quality. The median age of the entire cohort was 68 years. The clinicopathological characteristics of the FIGO III–IV group (*n* = 141) are presented in Table 1. Within this group, FIGO III cases predominated (*n* = 125; 88.7%), while FIGO IV accounted for 11.3% (*n* = 16). Endometrioid histotype was found in 76.0% (*n* = 98/129), grade G1–G2 in 58.1% (*n* = 75/129), and substantial LVSI in 37.2% (*n* = 48/129). Lymph node metastases were confirmed in 34.9% (*n* = 45/129), and distant metastases in 10.9% (*n* = 14/129). Histopathological data were available for *n* = 129 (MC, *n* = 12, reported separately; see Table 1 footnote).

### 3.2. Distribution of Molecular Subtypes by FIGO Stage

**Figure 1:**
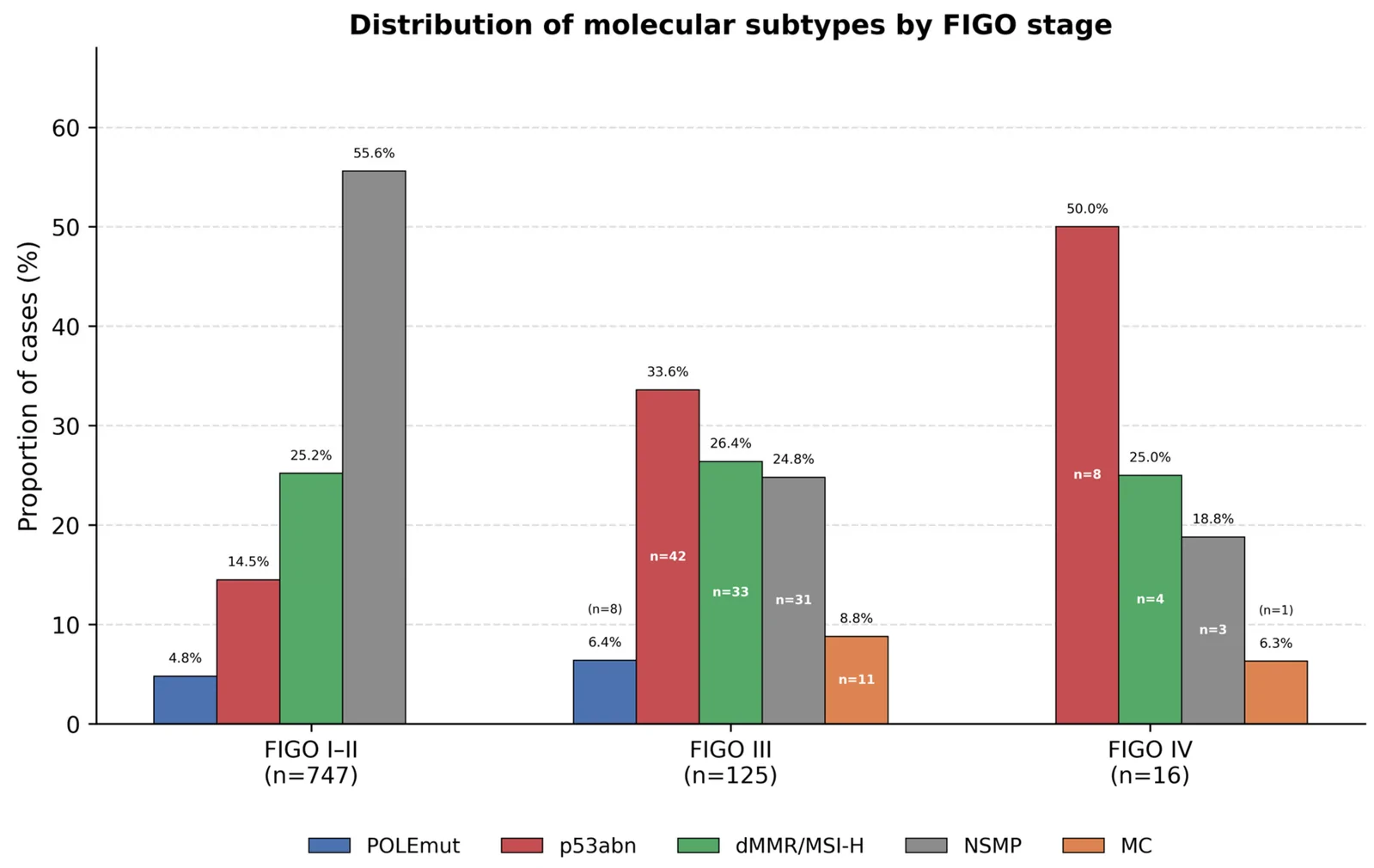
Distribution of molecular subtypes by FIGO stage (percentage among cases with a known subtype). The proportion of p53abn increases with advancing stage (14.5% → 33.6% → 50.0%; I–II vs. III–IV *p* < 0.001), while NSMP decreases (55.6% → 24.8% → 18.8%; I–II vs. III–IV *p* < 0.001), with a stable proportion of dMMR/MSI-H (I–II vs. III–IV *p* = 0.83) and no significant change in POLEmut (I–II vs. III–IV *p* = 0.67). Multiple-classifier (MC) cases are shown separately for FIGO III and IV; MC cases were not reported separately in FIGO I–II because overlapping tumours were classified according to the ProMisE hierarchy.

Table 2 shows the distribution of molecular subtypes across FIGO stages I–IV (known subtype in 888 patients). p53abn tumours showed a marked increase with advancing stage: 14.5% (FIGO I–II), 33.6% (FIGO III), and 50.0% (FIGO IV); the FIGO I–II vs. III–IV comparison was highly significant (Fisher *p* < 0.001) (Figure 1). NSMP predominated in early stages (55.6% in FIGO I–II), decreasing markedly in advanced stages (24.8% FIGO III; 18.8% FIGO IV; I–II vs. III–IV *p* < 0.001). In contrast, the frequency of POLEmut did not differ significantly between early and advanced stage: 4.8% (FIGO I–II), 6.4% (FIGO III), and 0% in FIGO IV (0/16; 95% Wilson CI 0.0–19.4%; I–II vs. III–IV *p* = 0.67; FIGO III vs. IV: Fisher *p* = 0.60). The absence of POLEmut in FIGO IV therefore represents an exploratory finding of limited statistical power owing to the small size of the FIGO IV subgroup (*n* = 16). Direct FIGO III vs. IV comparisons for individual subtypes did not reach significance (lowest *p* = 0.27, for p53abn). dMMR/MSI-H remained stable across all stages (I–II vs. III–IV *p* = 0.83; III vs. IV *p* = 1.00). Expressed as effect measures, the odds of advanced stages (III–IV vs. I–II) were more than three-fold higher for p53abn (OR 3.25; 95% CI 2.18–4.86) and reduced for NSMP (OR 0.25; 0.17–0.38), whereas no significant association with stage was observed for dMMR/MSI-H (OR 1.06; 0.70–1.59) or POLEmut (OR 1.19; 0.54–2.61) (Table 3). Of 225 dMMR/MSI-H cases in the entire cohort, 188 (83.6%) occurred in stages I–II, and 37 in stages III–IV.

### 3.3. POLEmut in FIGO III vs. FIGO IV—An Exploratory Observation

Table 4 compares the frequency of POLEmut in FIGO III and FIGO IV. POLEmut was identified in 6.4% of FIGO III cases (8/125; 95% Wilson CI 3.3–12.1%) and in no FIGO IV cases (0/16; 95% CI 0.0–19.4%; non-significant difference, Fisher *p* = 0.60).

### 3.4. Histopathological Correlations in FIGO III–IV

Table 5 presents the histopathological correlations of molecular subtypes in the FIGO III–IV group (*n* = 129; MC excluded). p53abn tumours showed a significant association with non-endometrioid histology (46.0%, *p* < 0.001) and grade G3 (60.0%, *p* = 0.008 for the difference between subtypes). Notably, no POLEmut case in FIGO III–IV showed non-endometrioid histotype (0/8), and the proportion of G3 was only 25.0%—the lowest among all subtypes. dMMR/MSI-H was characterised by the numerically highest proportion of substantial LVSI (45.9%), although the difference between subtypes did not reach statistical significance (*p* = 0.242). The highest proportion of lymph node metastases was observed in p53abn (42.0%) and dMMR/MSI-H (35.1%); for POLEmut it was 25.0% (non-significant difference between subtypes, *p* = 0.492). Distant metastases were most frequent in p53abn (16.0%); no case of distant metastasis was recorded in POLEmut (0/8; non-significant difference between subtypes, *p* = 0.609).

## 4. Discussion

### 4.1. p53abn as a Marker of Advanced Disease and a Determinant of Aggressive Treatment

The predominance of the p53abn subtype in FIGO III–IV (35.5%), with an increasing proportion up to 50.0% in FIGO IV, is consistent with the literature. Bosse et al. showed that 5-year overall survival (OS) for p53abn is 55%, versus 89% for POLEmut, 75% for dMMR/MSI-H, and 69% for NSMP, with p53 status remaining an independent prognostic factor for worse recurrence-free survival (RFS) [12]. Luzarraga Aznar et al. further characterised the recurrence pattern: distant metastases in 28.4% and peritoneal recurrences in 21.1% of patients with p53abn—compared with predominantly local recurrences in dMMR/MSI-H and only 2.1% overall recurrences in POLEmut [4].

The highest rate of lymph node metastases in p53abn (42.0%) and distant metastases (16.0%) in the present cohort confirms the aggressive phenotype of this subtype, although the differences in these features between subtypes did not reach statistical significance (*p* = 0.492 and *p* = 0.609, respectively). The absence of non-endometrioid histotype and distant metastases in the POLEmut subgroup (0/8 for both features) contrasts sharply with the p53abn profile.

For p53abn, ESGO/ESTRO/ESP guidelines recommend chemotherapy (carboplatin + paclitaxel) combined with radiotherapy (EBRT ± VBT) [1]. Results from the PORTEC-3 trial, confirmed in its 10-year follow-up, indicate that the benefit of combined chemoradiotherapy in molecular high-risk groups is most pronounced in the p53abn subtype, for both OS and RFS [13]. Jamieson et al. make the key observation that the benefit of adjuvant chemotherapy is limited to p53abn and does not extend to dMMR/MSI-H [14].

Data from Caiazzo et al., from a cohort of advanced EC, confirm the predominance of p53abn in FIGO IV [5].

### 4.2. dMMR/MSI-H—A Stable Candidate for Immunotherapy Regardless of Stage

In contrast to p53abn, whose frequency increased markedly with advancing disease, the distribution of dMMR/MSI-H remained stable regardless of FIGO stage. The stable frequency of dMMR/MSI-H across all FIGO stages—from 25.2% in FIGO I–II to 25.0% in FIGO IV (I–II vs. III–IV *p* = 0.83; III vs. IV *p* = 1.00)—is a key finding of this study. Although this proportion largely reflects the predominance of early-stage disease in the overall cohort, the stable distribution of dMMR/MSI-H across FIGO stages supports routine, stage-independent MMR testing, in agreement with current ESGO and NCCN recommendations. dMMR/MMR status is an important biomarker for immunotherapy eligibility in accordance with current clinical indications. The RUBY trial demonstrated a significant benefit of dostarlimab in patients with primary advanced or recurrent endometrial cancer and dMMR/MSI-H, encompassing both progression-free survival (PFS) and overall survival [15,16]. A benefit of adding a checkpoint inhibitor to chemotherapy was also shown in the non-MMRd population in the RUBY and NRG-GY018 trials, extending the role of immunotherapy beyond mismatch-repair-deficient tumours [15,17].

dMMR/MSI-H was characterised by the numerically highest proportion of substantial LVSI (45.9%), although the difference between subtypes did not reach statistical significance (*p* = 0.242). Data from the literature indicate that the prognostic significance of LVSI may depend on the molecular context and should not be interpreted in isolation [18,19,20,21,22,23].

### 4.3. POLEmut in FIGO III—An Exploratory Observation and Implications for De-Escalation

The absence of POLEmut in the FIGO IV group—given its presence in FIGO III—represents a hypothesis-generating observation of limited statistical power, requiring prospective validation. The frequency of POLEmut itself does not differ significantly between early and advanced stage (I–II vs. III–IV *p* = 0.67), and should therefore not be interpreted as a significant stage effect; what is significant is only the overall subtype distribution, dominated by the increase in p53abn. The histopathological profile of POLEmut in FIGO III–IV remains consistent with the favourable biology of this subtype (100% endometrioid histotype, 0% distant metastases), consistent with its well-documented favourable immunological profile [24,25].

Despite its favourable biological profile, 25.0% of POLEmut tumours in FIGO III–IV showed regional lymph node involvement (2/8), indicating that the presence of a POLE mutation does not preclude regional disease spread. The ESGO–ESTRO–ESP 2025 guidelines permit de-escalation only on an individual (case-by-case) basis within a multidisciplinary team (MDT) and after full staging, without a definitive recommendation owing to insufficient prospective data [1].

### 4.4. NSMP—Lack of Molecular Targets and Directions for Substratification

Whereas the molecular profiles of p53abn, dMMR/MSI-H, and POLEmut are each associated with specific treatment implications, NSMP remains the most heterogeneous and biologically least well-defined group of endometrial cancer, lacking specific molecular targets. A growing body of evidence indicates that absence of ER expression is associated with a higher risk of recurrence and less favourable prognosis, identifying a biologically more aggressive NSMP subgroup [26,27,28]. The prognostic significance of ER in this group has been confirmed by Perrone et al. [26], Aro et al. [27], and Vrede et al. [28]. In accordance with ESGO 2025 recommendations, ER/PR assessment may support further risk stratification within NSMP, while also enabling identification of patients with ER-positive tumours who may potentially benefit from hormone therapy [1]. ER/PR assessment was not performed routinely in our cohort (2022–2025), which represents a limitation of this study.

### 4.5. Implications for Clinical Practice

Current ESGO 2025 and NCCN guidelines indicate chemoimmunotherapy as the first-line standard of care for advanced endometrial cancer, with the choice of strategy depending on MMR/MSI and HER2 (human epidermal growth factor receptor 2) status [1,15,16,17,29,30,31,32,33]. The results of our cohort confirm the clinical utility of routine molecular profiling, as more than half of FIGO III–IV cases (61.7%) belonged to subtypes for which distinct treatment strategies are recommended (dMMR/MSI-H or p53abn), which may influence the choice of systemic therapy.

The treatment implications in Table 6 are based on extrapolation from randomised trials (RUBY, NRG-GY018, PORTEC-3) to the molecular profile of the present cohort—this study does not provide direct data on survival or treatment response.

Table 6 summarises the treatment implications of each molecular subtype in the context of ESGO/ESTRO/ESP guidelines, including the 2025 update.

### 4.6. Study Limitations

This study is retrospective and descriptive in nature and does not include OS or PFS data—prognostic conclusions are based on data from the literature. Interpretation of the observation regarding POLEmut in FIGO IV is limited by the small size of this subgroup (*n* = 16). Restaging according to the FIGO 2023 classification was not feasible owing to incomplete availability of the required pathological parameters in historical records. Because FIGO 2023 incorporates molecular features into stage assignment, this inability to restage the cohort may affect direct comparison with contemporary molecularly integrated stage groups in selected cases. ER/PR assessment was not performed routinely during the study period (2022–2025), precluding substratification of NSMP. The absence of multivariable analysis precludes identification of independent predictors of advanced stage. The lack of central pathological review may affect the consistency of classification between centres. The cohort included only patients treated surgically, which may lead to underrepresentation of inoperable FIGO IVB cases and affect the observed subtype distribution in stage IV.

## 5. Conclusions

1. Advanced endometrial cancer (FIGO III–IV) is characterised by predominance of the p53abn subtype (35.5%), whose proportion increases with stage (I–II vs. III–IV *p* < 0.001), reflecting an aggressive phenotype prone to systemic spread.

2. Tumours with mismatch repair deficiency (dMMR/MSI-H) show a stable frequency across all FIGO stages (I–II vs. III–IV *p* = 0.83), supporting routine MMR testing in all patients with endometrial cancer (83.6% of dMMR/MSI-H cases occurred in FIGO I–II in this cohort, largely reflecting the predominance of early-stage disease), in agreement with current ESGO and NCCN recommendations, and enabling identification of patients who may benefit from immunotherapy (dostarlimab/pembrolizumab) in accordance with current clinical indications.

3. The frequency of POLEmut does not differ significantly between early and advanced stage (I–II vs. III–IV *p* = 0.67); its absence in FIGO IV in this cohort represents an exploratory finding of limited statistical power, which should be interpreted with caution. In accordance with current ESGO guidelines, treatment de-escalation in patients with POLEmut and advanced endometrial cancer is not a standard of care and may be considered only individually, following multidisciplinary team assessment.

4. The histopathological profile of POLEmut in FIGO III–IV (100% endometrioid, 0% distant metastases) contrasts sharply with the aggressive profile of p53abn (46.0% non-endometrioid, 16.0% distant metastases).

## Figures and Tables

**Table 1:** Baseline clinicopathological characteristics of the advanced cohort (FIGO III–IV; *n* = 141 with known molecular subtype).

Variable/Category	N (%)
**FIGO stage (2009)**	
FIGO III	125 (88.7%)
FIGO IV	16 (11.3%)
**Molecular subtype**	
p53abn	50 (35.5%)
dMMR/MSI-H	37 (26.2%)
NSMP	34 (24.1%)
Multiple classifier (MC)	12 (8.5%)
POLEmut	8 (5.7%)
**Age at surgery (years)**	
<60	39 (27.7%)
60–70	50 (35.5%)
>70	52 (36.9%)
**Histotype (*n* = 129)**	
Endometrioid	98 (76.0%)
Non-endometrioid	31 (24.0%)
**Tumour grade (*n* = 129)**	
G1–G2	75 (58.1%)
G3	54 (41.9%)
**LVSI (*n* = 129)**	
Absent/focal	81 (62.8%)
Substantial	48 (37.2%)
**Lymph node metastases (*n* = 129)**	
Absent	84 (65.1%)
Present	45 (34.9%)
**Distant metastases (*n* = 129)**	
Absent	115 (89.1%)
Present	14 (10.9%)
**Total (FIGO III–IV, known molecular subtype)**	**141 (100%)**

Age is reported for the entire FIGO III–IV subgroup (*n* = 141). Histopathological comparative analyses were performed on *n* = 129 after excluding MC cases (*n* = 12), which are reported descriptively; *n* = 129 reflects the analytical subset for histopathological features, not a difference in data availability. Percentages for these features were calculated within *n* = 129. LVSI—lymphovascular space invasion; POLEmut—POLE-mutated; dMMR/MSI-H—deficient mismatch repair; MSI-H—microsatellite instability-high; p53abn—p53 abnormal; NSMP—no specific molecular profile; MC—multiple classifier. FIGO 2009 staging.

**Table 2:** Distribution of molecular subtypes by FIGO stage (known subtype, N = 888).

FIGO Stage	N	POLEmut	p53abn	dMMR/MSI-H	NSMP	MC
I–II	747	36 (4.8%)	108 (14.5%)	188 (25.2%)	415 (55.6%)	—
III	125	8 (6.4%)	42 (33.6%)	33 (26.4%)	31 (24.8%)	11 (8.8%)
IV	16	0 (0.0%)	8 (50.0%)	4 (25.0%)	3 (18.8%)	1 (6.3%)
III–IV (combined)	141	8 (5.7%)	50 (35.5%)	37 (26.2%)	34 (24.1%)	12 (8.5%)
**Overall (known subtype)**	**888**	**44 (5.0%)**	**158 (17.8%)**	**225 (25.3%)**	**449 (50.6%)**	**12 (1.4%)**
*p* (I–II vs. III–IV) †	—	0.67	**<0.001**	0.83	**<0.001**	n/a ‡
*p* (III vs. IV) †	—	0.60	0.27	1.00	0.76	1.00

A conclusive molecular subtype was obtained in 888 of 915 patients (27 cases without a conclusive subtype were excluded from subtype-specific analyses). MC—multiple classifier; ProMisE/WHO hierarchy: POLEmut > dMMR > p53abn > NSMP. ‡ In the FIGO I–II group, tumours with overlapping molecular features were classified per the ProMisE hierarchy into one of the four canonical subtypes and were not reported separately as MC; therefore, the I–II vs. III–IV comparison for MC is not interpretable and was omitted. † Two-sided Fisher’s exact test. The increase in p53abn and decrease in NSMP in advanced stages were both significant (*p* < 0.001); the frequencies of POLEmut and dMMR did not differ significantly between stages. FIGO 2009 staging.

**Table 3:** Odds ratio for advanced stage (FIGO III–IV vs. I–II) by molecular subtype.

Molecular Subtype	OR (III–IV vs. I–II)	95% CI	*p* †
POLEmut	1.19	0.54–2.61	0.67
p53abn	3.25	2.18–4.86	**<0.001**
dMMR/MSI-H	1.06	0.70–1.59	0.83
NSMP	0.25	0.17–0.38	**<0.001**

OR—odds ratio for advanced stage (FIGO III–IV vs. I–II), each subtype versus all other subtypes combined (reference category); 95% CI by the Woolf (logit) method. † *p*-value—two-sided Fisher’s exact test (identical to the I–II vs. III–IV comparison in Table 2). MC was omitted because tumours with overlapping molecular features were not reported separately in FIGO I–II but were assigned to one of the four canonical subtypes according to the ProMisE hierarchy. OR > 1 indicates enrichment in advanced stages, OR < 1—depletion. FIGO 2009 staging.

**Table 4:** Frequency of POLEmut in FIGO III vs. FIGO IV—an exploratory observation.

FIGO Stage	POLEmut *n*/N	%	95% CI (Wilson)	*p* †
FIGO III	8/125	6.4%	3.3–12.1%	0.60
FIGO IV	0/16	0.0%	0.0–19.4%	—

95% CI—Wilson (score) confidence interval, appropriate for small *n* and a zero-count cell in FIGO IV. † Two-sided Fisher’s exact test; the *p*-value refers to the III vs. IV comparison overall and is reported once, in the FIGO III row. The absence of POLEmut in FIGO IV (0/16) is an exploratory observation; prospective validation is required. The analysis is underpowered owing to *n* = 16 in FIGO IV.

**Table 5:** Histopathological features of molecular subtypes in advanced endometrial cancer (FIGO III–IV; *n* = 129, MC excluded).

Feature	p53abn (*n* = 50)	dMMR/MSI-H (*n* = 37)	NSMP (*n* = 34)	POLEmut (*n* = 8)	*p* †
**Histotype**					**<0.001**
Endometrioid	27 (54.0%)	32 (86.5%)	31 (91.2%)	8 (100.0%)	
Non-endometrioid	23 (46.0%)	5 (13.5%)	3 (8.8%)	0 (0.0%)	
**Tumour grade**					**0.008**
G1–G2	20 (40.0%)	24 (64.9%)	25 (73.5%)	6 (75.0%)	
G3	30 (60.0%)	13 (35.1%)	9 (26.5%)	2 (25.0%)	
**LVSI**					**0.242**
Absent/focal	30 (60.0%)	20 (54.1%)	24 (70.6%)	7 (87.5%)	
Substantial	20 (40.0%)	17 (45.9%)	10 (29.4%)	1 (12.5%)	
**Lymph node metastases**					**0.492**
Absent	29 (58.0%)	24 (64.9%)	25 (73.5%)	6 (75.0%)	
Present	21 (42.0%)	13 (35.1%)	9 (26.5%)	2 (25.0%)	
**Distant metastases**					**0.609**
Absent	42 (84.0%)	34 (91.9%)	31 (91.2%)	8 (100.0%)	
Present	8 (16.0%)	3 (8.1%)	3 (8.8%)	0 (0.0%)	

Values are reported as *n* (%) within each molecular subtype column; restricted to FIGO III–IV cases with a known subtype, excluding MC (*n* = 12) (column totals: 50 + 37 + 34 + 8 = 129). † Fisher–Freeman–Halton exact test (2 × 4, two-sided), appropriate for each variable, as expected cell counts < 5 occurred in every comparison (mainly owing to POLEmut, *n* = 8). LVSI—lymphovascular space invasion.

**Table 6:** Treatment implications of molecular subtypes in advanced endometrial cancer (FIGO III–IV; *n* = 141).

Subtype (% of FIGO III–IV)	Biological Characteristics	Treatment Implications	Key Evidence/Guidelines
dMMR/MSI-H—26.2% (*n* = 37)	Hypermutated, immunologically active phenotype; high neoantigen burden; frequency stable across all FIGO stages (I–II vs. III–IV *p* = 0.83); strong tumour-infiltrating lymphocyte (TIL) infiltration.	dMMR/MMR status identifies patients eligible for immunotherapy in appropriate clinical situations, in accordance with current guidelines; dostarlimab + carboplatin/paclitaxel (RUBY); pembrolizumab + chemotherapy (NRG-GY018); pembrolizumab in MSI-H disease after prior treatment (KEYNOTE-158). Universal MMR testing in all patients with endometrial cancer.	RUBY; NRG-GY018; ESGO 2025.
p53abn—35.5% (*n* = 50)	Copy-number-high (serous-like) phenotype; aggressive profile; predominant subtype in FIGO IV (50.0%); enriched in advanced versus early stages (*p* < 0.001); highest proportion of non-endometrioid histology (46.0%) and grade G3 (60.0%).	Platinum-based chemotherapy combined with radiotherapy (EBRT ± VBT), PORTEC-3 regimen for high-risk/advanced disease; anti-HER2 therapy in HER2-positive serous carcinoma; the role of ICIs in p53abn tumours without concurrent dMMR/MSI-H remains less well-established than in dMMR/MSI-H; enrolment in the RAINBO trial (p53abn-RED arm) is encouraged.	PORTEC-3; ESGO 2025.
POLEmut—5.7% (*n* = 8)	Ultramutated (>100 mut/Mb), favourable immunological biology; not observed in FIGO IV in this cohort (0/16; 95% CI 0.0–19.4%); lowest proportion of non-endometrioid histology (0%) and distant metastases (0%); high TIL density.	Management is generally consistent with standard stage-based protocols; treatment intensity is determined after complete staging and multidisciplinary team (MDT) discussion; de-escalation is not currently a standard of care in patients with advanced EC and should be considered individually within the MDT; caution is warranted in MC tumours, where POLEmut co-occurs with p53abn. POLEmut does not preclude advanced disease.	RAINBO; ESGO 2025.
NSMP—24.1% (*n* = 34)	Heterogeneous group with intermediate risk; frequent ER/PR expression; depleted in advanced versus early stages (*p* < 0.001); intermediate proportion of G3 (26.5%) and N+ (26.5%).	Platinum-based chemotherapy ± radiotherapy depending on FIGO stage; hormone therapy in selected ER-positive cases; lenvatinib + pembrolizumab in advanced/recurrent disease after prior treatment (KEYNOTE-775). The 2025 update divides NSMP into ER-positive/low-grade and ER-negative/high-grade subgroups for risk stratification.	KEYNOTE-775; ESGO 2025.

Data are restricted to FIGO III–IV cases with a known molecular subtype (*n* = 141). Frequencies are reported as % of the FIGO III–IV subgroup. ICI—immune checkpoint inhibitor; dMMR/MSI-H—deficient mismatch repair; MSI-H—microsatellite instability-high; TIL—tumour-infiltrating lymphocytes; EBRT—external beam radiotherapy; VBT—vaginal brachytherapy; ER/PR—oestrogen/progesterone receptor; MDT—multidisciplinary team. Recommendations are consistent with the ESGO–ESTRO–ESP 2025 guidelines; management of individual patients requires MDT assessment.

## Data Availability

The data presented in this study are available on request from the corresponding author due to restrictions related to patient privacy and compliance with GDPR.
